# Isolated Fungal Sphenoid Sinusitis With Cavernous Sinus Thrombophlebitis: A Case Report

**DOI:** 10.7759/cureus.25034

**Published:** 2022-05-16

**Authors:** Maria Clarissa Nunez, Ma. Luisa Gwenn P Tiongson

**Affiliations:** 1 Clinical Neurosciences, University of the East Ramon Magsaysay Memorial Medical Center, Quezon City, PHL

**Keywords:** fungal sinusitis, rhinocerebral, sphenoid sinusitis, mucormycosis, cavernous sinus thrombophlebitis

## Abstract

Cavernous sinus thrombophlebitis is a rare, potentially life-threatening, condition that is most often caused by gram-negative bacteria and, to a lesser extent, fungi. *Mucor* is an opportunistic fungus that frequently affects patients with a weak immune system. We describe a case of an adult female without diabetes who developed *Mucor* sphenoid sinusitis causing cavernous sinus thrombophlebitis. The patient presented with headache, diplopia, and right lateral rectus palsy. Cranial magnetic resonance imaging (MRI) showed abnormal prominent enhancement involving the cavernous sinuses associated with interspersed internal non-enhancing components indicating bilateral cavernous sinus thrombophlebitis and exuberant inspissated secretions within the left sphenoid sinus. After administering enoxaparin and intravenous antibiotics, the patient underwent endoscopic transnasal sphenoidotomy with nasal polypectomy. Culture results showed growth of mucor, for which the patient received itraconazole. Thereafter, complete resolution of headache, diplopia, and right lateral rectus palsy was observed. On follow-up, no residual neurologic deficits were noted. The repeat cranial MRI showed no abnormality involving the cavernous sinuses, with no evidence of cavernous sinus thrombophlebitis and normal paranasal sinuses. While a few case reports have been available on cavernous sinus thrombophlebitis caused by fungal sphenoid sinusitis with *Mucor* as the primary organism, none have involved immunocompetent individuals.

## Introduction

Cavernous sinus thrombosis is more frequently caused by gram-negative bacteria and less commonly by fungi. For bacterial and fungal causes, the most common organisms are *Staphylococcus aureus* [1] and *Aspergillus fumigatus* [2], respectively. *Mucor* is a rare, angioinvasive opportunistic fungal pathogen affecting the lungs, sinuses, and brain [3]. This condition has been associated with high morbidity and mortality due to the angioinvasive property of the fungus, which causes vascular occlusion, consequently resulting in extensive tissue necrosis [4]. Patients with poorly controlled diabetes, those who had undergone transplantation, those with kidney failure, and those who are immunocompromised are at a high risk of developing mucormycosis. The most common presenting symptoms are headache, fever, periorbital swelling, and ophthalmoplegia [5]. The prevalence of cavernous sinus thrombosis has diminished due to the accessibility of medications; however, early recognition is vital considering the high mortality rate associated with delayed treatment. While some published data is available regarding *Mucor* sphenoid sinusitis as the cause of cavernous sinus thrombophlebitis, this is the first description of an immunocompetent patient with sphenoid sinusitis and cavernous sinus thrombosis secondary to *Mucor*, to the best of our knowledge. Hence, we present an unusual case of an immunocompetent patient who developed cavernous sinus thrombosis secondary to *Mucor *sphenoid sinusitis.

## Case presentation

**Figure 1:**
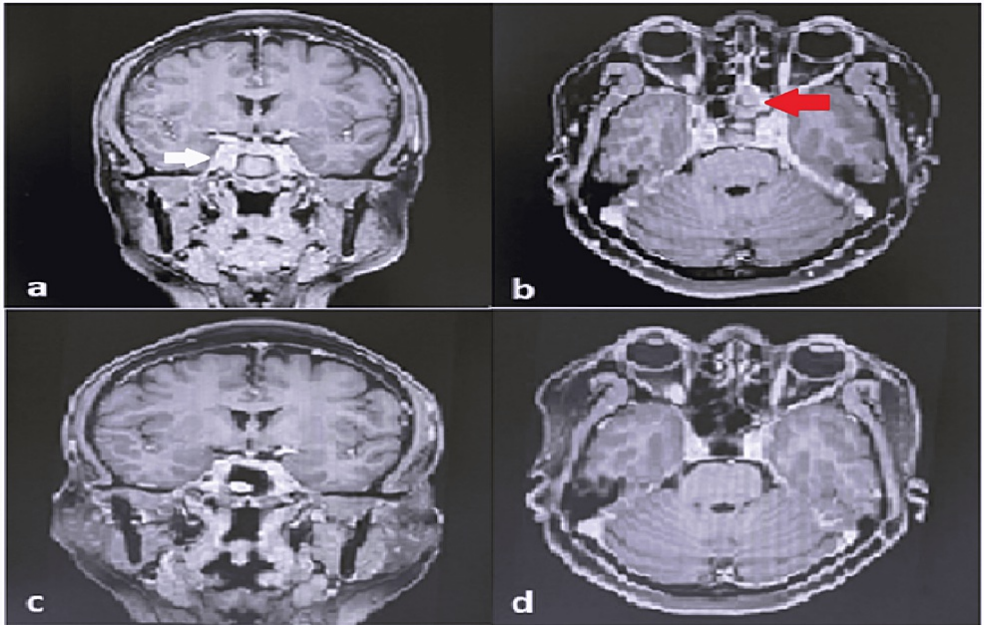
Plain and contrast-enhanced cranial magnetic resonance imaging of the patient (a) T1 post-contrast coronal cut shows prominent abnormal enhancement involving the cavernous sinuses accompanied by interspersed internal non-enhancing abnormalities with an asymmetric degree of involvement of the right cavernous sinus (white arrow); (b) T1 post-contrast shows the inspissated secretions within the left sphenoid sinus. (red arrow); (c) T1 post contrast coronal cut. A follow-up study showed no abnormality involving the cavernous sinuses. The cavernous sinuses demonstrate normal flow and contrast enhancement; (d) The T1 post-contrast follow-up study demonstrates the absence of left sphenoid sinus secretions.

A 53-year-old female patient without diabetes, presented with a right temporoparietal headache lasting for three weeks, throbbing and associated with vomiting and photophobia. She was initially diagnosed to have a migraine headache, for which she was prescribed pain medications that provided temporary relief. Three days prior to admission, she developed binocular doubling of vision. Past medical history includes hypertension with a known allergy to chickens, no malignancy, no previous surgeries, and no history of diabetes or malignancy in the family. Upon examination, vital signs were stable and neurologic examination showed right lateral rectus palsy, no anosmia, no visual field cuts, no hemorrhages or exudates seen on fundoscopy, pupils are 3 mm briskly reactive to light, the primary gaze is midline, no ptosis, no facial asymmetry, no focal motor, and sensory deficit. MRI (Figure 1a) showed abnormal prominent enhancement involving the cavernous sinuses associated with interspersed internal non-enhancing components, which indicates bilateral cavernous sinus thrombophlebitis. Exuberant inspissated secretions within the left sphenoid sinus were noted (Figure 1b). Complete blood count showed hemoglobin of 123 g/dL, hematocrit of 38 %, white blood cell count of 8.3 X10^9/L, neutrophils of 61%, lymphocytes 37%, monocytes 0, eosinophils 2, basophils 0, and platelet count of 316 X10^9/L, fasting blood glucose of 99 mg/dL, protime of 10.9 with control 12.0, international normalized ratio (INR) of 0.90% activity of 104, and activated partial thromboplastin time of 25.4 with control of 30.0. The patient was started on enoxaparin 40mg subcutaneously every 12 hours, ceftriaxone 2g intravenously every 24 hours, and metronidazole 500 mg intravenously every six hours, and was referred to the Otorhinolaryngology for co-management. Following endoscopic transnasal sphenoidotomy with nasal polypectomy, fungal culture results showed growth of *Mucor* with sensitivity to amphotericin B, fluconazole, flucytosine, and voriconazole. The antibiotics were de-escalated, and fluconazole 100 mg/tablet, one tablet once daily was started. Thereafter, resolution of the headache, diplopia, and right lateral rectus palsy was observed postoperatively. Prior to discharge, fluconazole was shifted to itraconazole 200 mg/tablet, one tablet twice a day for six weeks, whereas enoxaparin was shifted to warfarin 5 mg/tablet, one tablet once daily. The patient was discharged asymptomatic on day 11 of being admitted to the hospital, and oral anticoagulation was eventually discontinued after three weeks.

Upon follow-up after one month, a repeat MRI (Figure 1c) showed no abnormality involving the cavernous sinuses, with no evidence of cavernous sinus thrombophlebitis. MR venogram demonstrated no evidence of veno-occlusive disease, with a normal MRI (Figure 1d) of the paranasal sinuses. No evidence of a contrast-enhancing abnormality was observed within the nasal cavities and paranasal sinuses.

## Discussion

Despite the reports on the occurrence of fungal sphenoid sinusitis leading to cavernous sinus thrombophlebitis, a wide literature search did not yield any publication on mucormycosis in an immunocompetent patient without diabetes. The anatomical importance of the sphenoid sinus is due to its proximity and location between both cavernous sinuses [6]. The proposed mechanism involves hematogenous spread causing the fungi to adhere to the endothelium of neighboring blood vessels, resulting in thrombus formation. Given the lack of valves in the dural sinus system, a multidirectional flow through the emissary veins into and out of the cavernous sinus allows the thrombus to propagate into the dural system [5].

The anatomy and structures within the cavernous sinus are of clinical importance. Its close relationship with cranial nerves III, IV, V, and VI, as well as the horizontal branch of the internal carotid artery, explains the presenting signs and symptoms, namely headache, diplopia, and right lateral rectus palsy. Diplopia and right lateral rectus palsy were caused by the local compression and inflammation of the abducens nerve.

This case report highlights an unusual occurrence of cavernous sinus thrombophlebitis caused by *Mucor* sphenoid sinusitis in an immunocompetent patient. Poorly controlled diabetes remains the leading risk factor for mucormycosis. While some reports have included patients with poor control of diabetes [7-10], others have included patients with diabetic ketoacidosis upon admission [3,11,12]. Patients with end-stage renal disease undergoing dialysis and receiving deferoxamine and immunosuppressant drugs are also at risk. Johnson et al. reported a case of an adult male patient with end-stage renal disease undergoing dialysis while on deferoxamine [13], whereas Haber et al. reported an adult diabetic female using immunosuppressant drugs after kidney transplantation [14]. Chronic complications of fungal sinus infections include cavernous sinus thrombosis, pituitary invasion, internal carotid artery, ophthalmic, and retinal artery thrombosis [2,3]. Fungi produce thrombosis by invading the vasculature, resulting in extensive coagulative necrosis and gangrene [13]. Aside from cavernous sinus thrombosis, some develop other complications, such as occlusion of the internal carotid artery [3,10], bilateral central retinal artery occlusion [13], and cavernous thrombosis with middle cerebral artery occlusion [8]. In our patient, the magnetic resonance angiography and magnetic resonance venography did not show any occlusion.

Treatment included early surgical intervention and control of the source of infection with an empiric antibiotic regimen [15], which lasted at least three to four weeks in accordance with other intravascular infections [16]. Considering the rarity of this disease, hardly any randomized control trials have determined the efficacy of anticoagulation use. There is still no consensus regarding the duration, type, and dose of anticoagulation, which has been suggested to improve outcomes and halt the progression of the thrombus. In most studies, amphotericin B had been the frequently used antifungal treatment but some reports also showed successful outcome with posaconazole and itraconazole. The first-line antifungal treatment is amphothericin B and a step-down treatment with posaconazole or isavuconazole. There are few published articles about the use of itraconazole as a potential agent for the treatment of mucormycosis [17]. Given the lack of clinical trials and limited data to determine the duration of treatment and follow-up imaging, there are no specific recommendations on these. However, a few published reports suggested to continue with the antifungal and anticoagulant treatment until a resolution is seen on imaging.

Our case is unique considering the lack of other publications regarding the successful itraconazole treatment of mucormycosis causing cavernous sinus thrombosis in an immunocompetent patient without diabetes.

## Conclusions

Cavernous sinus thrombophlebitis caused by mucormycosis is a rare disease with high morbidity and mortality. Despite its increased frequency in patients with diabetes, malignancies, and immunosuppressive drug intake, this condition can still occur in patients without comorbidities. Therefore, it should still be considered as part of the differential diagnosis for patients with clinical signs and symptoms of cavernous sinus thrombosis, given the importance of early recognition and treatment for a good outcome. 
